# Supplementary material for: Modeling glioblastoma heterogeneity as a dynamic network of cell states
Source: Mol Syst Biol. 2021 Sep 16;17(9):e10105. doi: 10.15252/msb.202010105 (PMC8444284; doi:10.15252/msb.202010105)
Supplement: Supplementary file 5 — Source Data for Figure 3 [file MSB-17-e10105-s001.zip › Figure3A_sourcedata/GSEA_3065/nosigdb_state6.GseaPreranked.1622718443993/OLSEN_MESENCHYMAL.html]

Details for gene set OLSEN\_MESENCHYMAL[GSEA]

|  || Dataset | state6 |
| Phenotype | NoPhenotypeAvailable |
| Upregulated in class | na\_neg |
| GeneSet | OLSEN\_MESENCHYMAL |
| Enrichment Score (ES) | -0.5458282 |
| Normalized Enrichment Score (NES) | -2.0434506 |
| Nominal p-value | 0.0 |
| FDR q-value | 1.2007895E-4 |
| FWER p-Value | 0.002 |
Table: GSEA Results Summary

  

Fig 1: Enrichment plot: OLSEN\_MESENCHYMAL      
 Profile of the Running ES Score & Positions of GeneSet Members on the Rank Ordered List

  

| PROBE | GENE SYMBOL | GENE\_TITLE | RANK IN GENE LIST | RANK METRIC SCORE | RUNNING ES | CORE ENRICHMENT || 1 | RPLP1 |  |  | 6 | 0.329 | 0.0347 | No |
| 2 | RPL41 |  |  | 42 | 0.249 | 0.0574 | No |
| 3 | TPT1 |  |  | 48 | 0.247 | 0.0834 | No |
| 4 | FTL |  |  | 64 | 0.234 | 0.1068 | No |
| 5 | RPL32 |  |  | 69 | 0.231 | 0.1311 | No |
| 6 | RPS25 |  |  | 84 | 0.223 | 0.1535 | No |
| 7 | SERF2 |  |  | 97 | 0.215 | 0.1752 | No |
| 8 | TMSB4X |  |  | 162 | 0.187 | 0.1880 | No |
| 9 | ZFP36L1 |  |  | 266 | 0.159 | 0.1932 | No |
| 10 | EEF1D |  |  | 682 | 0.107 | 0.1572 | No |
| 11 | MT2A |  |  | 1097 | 0.081 | 0.1184 | No |
| 12 | ID3 |  |  | 1329 | 0.070 | 0.0994 | No |
| 13 | IFITM3 |  |  | 1398 | 0.068 | 0.0989 | No |
| 14 | GADD45B |  |  | 1497 | 0.064 | 0.0946 | No |
| 15 | LIMA1 |  |  | 1702 | 0.057 | 0.0773 | No |
| 16 | CAVIN1 |  |  | 1909 | 0.050 | 0.0591 | No |
| 17 | SELENOM |  |  | 2281 | 0.039 | 0.0208 | No |
| 18 | S100A11 |  |  | 2523 | 0.033 | -0.0033 | No |
| 19 | VIM |  |  | 2831 | 0.026 | -0.0357 | No |
| 20 | FN1 |  |  | 2839 | 0.026 | -0.0338 | No |
| 21 | TPM2 |  |  | 4007 | 0.001 | -0.1674 | No |
| 22 | JUNB |  |  | 4044 | -0.000 | -0.1715 | No |
| 23 | AEBP1 |  |  | 4222 | -0.004 | -0.1914 | No |
| 24 | S100A6 |  |  | 4326 | -0.005 | -0.2026 | No |
| 25 | FOS |  |  | 4536 | -0.010 | -0.2255 | No |
| 26 | S100A13 |  |  | 4664 | -0.012 | -0.2388 | No |
| 27 | TIMP3 |  |  | 5017 | -0.019 | -0.2772 | No |
| 28 | TAGLN |  |  | 5323 | -0.025 | -0.3094 | No |
| 29 | LHFPL6 |  |  | 5460 | -0.028 | -0.3220 | No |
| 30 | PPIC |  |  | 5491 | -0.028 | -0.3225 | No |
| 31 | UACA |  |  | 5586 | -0.030 | -0.3300 | No |
| 32 | IFITM2 |  |  | 5741 | -0.033 | -0.3440 | No |
| 33 | CST3 |  |  | 5892 | -0.036 | -0.3574 | No |
| 34 | C1R |  |  | 6276 | -0.044 | -0.3965 | No |
| 35 | EGR1 |  |  | 6519 | -0.050 | -0.4189 | No |
| 36 | CD99 |  |  | 6572 | -0.052 | -0.4192 | No |
| 37 | NNMT |  |  | 6762 | -0.058 | -0.4347 | No |
| 38 | IGFBP5 |  |  | 7011 | -0.065 | -0.4561 | No |
| 39 | IGFBP7 |  |  | 7318 | -0.075 | -0.4831 | No |
| 40 | NPC2 |  |  | 7426 | -0.079 | -0.4869 | No |
| 41 | CCDC80 |  |  | 7480 | -0.080 | -0.4844 | No |
| 42 | PRRX1 |  |  | 7482 | -0.081 | -0.4758 | No |
| 43 | COL14A1 |  |  | 8094 | -0.113 | -0.5337 | Yes |
| 44 | LY6E |  |  | 8146 | -0.117 | -0.5270 | Yes |
| 45 | LMNA |  |  | 8225 | -0.124 | -0.5226 | Yes |
| 46 | COL6A2 |  |  | 8253 | -0.126 | -0.5121 | Yes |
| 47 | SPARC |  |  | 8303 | -0.132 | -0.5035 | Yes |
| 48 | GSN |  |  | 8339 | -0.137 | -0.4928 | Yes |
| 49 | FSTL1 |  |  | 8373 | -0.143 | -0.4812 | Yes |
| 50 | COL5A1 |  |  | 8428 | -0.150 | -0.4713 | Yes |
| 51 | THY1 |  |  | 8454 | -0.153 | -0.4577 | Yes |
| 52 | COL5A2 |  |  | 8514 | -0.165 | -0.4468 | Yes |
| 53 | COL4A2 |  |  | 8525 | -0.168 | -0.4300 | Yes |
| 54 | PCOLCE |  |  | 8639 | -0.209 | -0.4205 | Yes |
| 55 | EMP1 |  |  | 8641 | -0.209 | -0.3982 | Yes |
| 56 | SERPINH1 |  |  | 8646 | -0.212 | -0.3759 | Yes |
| 57 | CALD1 |  |  | 8649 | -0.213 | -0.3533 | Yes |
| 58 | ANXA1 |  |  | 8651 | -0.213 | -0.3305 | Yes |
| 59 | COL4A1 |  |  | 8661 | -0.218 | -0.3081 | Yes |
| 60 | VCAN |  |  | 8664 | -0.219 | -0.2848 | Yes |
| 61 | PRSS23 |  |  | 8688 | -0.233 | -0.2624 | Yes |
| 62 | COL6A1 |  |  | 8698 | -0.238 | -0.2379 | Yes |
| 63 | ITM2B |  |  | 8705 | -0.242 | -0.2126 | Yes |
| 64 | B2M |  |  | 8718 | -0.258 | -0.1863 | Yes |
| 65 | TIMP1 |  |  | 8751 | -0.290 | -0.1588 | Yes |
| 66 | RRBP1 |  |  | 8768 | -0.320 | -0.1263 | Yes |
| 67 | NEAT1 |  |  | 8775 | -0.334 | -0.0910 | Yes |
| 68 | CD63 |  |  | 8776 | -0.335 | -0.0551 | Yes |
| 69 | POSTN |  |  | 8795 | -0.533 | 0.0001 | Yes |
Table: GSEA details [plain text format]

  

Fig 2: OLSEN\_MESENCHYMAL: Random ES distribution      
 Gene set null distribution of ES for **OLSEN\_MESENCHYMAL**
